# Strategies used to reduce harms associated with fentanyl exposure among rural people who use drugs: multi-site qualitative findings from the rural opioid initiative

**DOI:** 10.1186/s12954-024-01062-2

**Published:** 2024-08-24

**Authors:** Suzan M. Walters, Robin Baker, David Frank, Monica Fadanelli, Abby E. Rudolph, William Zule, Rob J. Fredericksen, Rebecca Bolinski, Adams L. Sibley, Vivian F. Go, Lawrence J. Ouellet, Mai T. Pho, David W. Seal, Judith Feinberg, Gordon Smith, April M. Young, Thomas J. Stopka

**Affiliations:** 1 Department of Population Health, New York University Grossman School of Medicine, New York, NY USA; 2 Learning Design and Innovation, Dartmouth College, Hanover, NH USA; 3 Department of Social and Behavioral Health, School of Global Public Health, New York University, New York, NY USA; 4 Rollins School of Public Health, Emory University, Atlanta, GA USA; 5 Department of Epidemiology and Biostatistics, Temple University College of Public Health, Philadelphia, PA USA; 6 RTI International, Research Triangle Park, NC USA; 7 Department of Medicine, University of Washington, Seattle, WA USA; 8 Southern Illinois University, Carbondale, IL USA; 9 Department of Health Behavior, Gillings School of Global Public Health, University of North Carolina-Chapel Hill, Chapel Hill, NC USA; 10 Division of Epidemiology and Biostatistics, School of Public Health, University of Illinois Chicago, Chicago, IL USA; 11 Department of Medicine, University of Chicago, Chicago, IL USA; 12 Social, Behavioral, and Population Sciences, Tulane University School of Public Health and Tropical Medicine, New Orleans, LA USA; 13 Departments of Behavioral Medicine and Psychiatry and Medicine/Infectious Diseases, School of Medicine, West Virginia University, Morgantown, WV USA; 14 Department of Epidemiology and Biostatistics, School of Public Health, West Virginia University, Morgantown, WV USA; 15 Department of Epidemiology and Environmental Health, University of Kentucky College of Public Health, Lexington, KY USA; 16 Department of Public Health and Community Medicine, Tufts University School of Medicine, Boston, MA USA

## Abstract

**Aim:**

Illicitly manufactured fentanyl and its analogs are the primary drivers of opioid overdose deaths in the United States (U.S.). People who use drugs may be exposed to fentanyl or its analogs intentionally or unintentionally. This study sought to identify strategies used by rural people who use drugs to reduce harms associated with unintentional fentanyl exposure.

**Methods:**

This analysis focused on 349 semi-structured qualitative interviews across 10 states and 58 rural counties in the U.S conducted between 2018 and 2020. Interview guides were collaboratively standardized across sites and included questions about drug use history (including drugs currently used, frequency of use, mode of administration) and questions specific to fentanyl. Deductive coding was used to code all data, then inductive coding of overdose and fentanyl codes was conducted by an interdisciplinary writing team.

**Results:**

Participants described being concerned that fentanyl had saturated the drug market, in both stimulant and opioid supplies. Participants utilized strategies including: (1) avoiding drugs that were perceived to contain fentanyl, (2) buying drugs from trusted sources, (3) using fentanyl test strips, 4) using small doses and non-injection routes, (5) using with other people, (6) tasting, smelling, and looking at drugs before use, and (7) carrying and using naloxone. Most people who used drugs used a combination of these strategies as there was an overwhelming fear of fatal overdose.

**Conclusion:**

People who use drugs living in rural areas of the U.S. are aware that fentanyl is in their drug supply and use several strategies to prevent associated harms, including fatal overdose. Increasing access to harm reduction tools (e.g., fentanyl test strips, naloxone) and services (e.g., community drug checking, syringe services programs, overdose prevention centers) should be prioritized to address the polysubstance-involved overdose crisis. These efforts should target persons who use opioids and other drugs that may contain fentanyl.

**Supplementary Information:**

The online version contains supplementary material available at 10.1186/s12954-024-01062-2.

## Introduction

The United States continues to experience the worst overdose epidemic in the world [1]. The COVID-19 pandemic has exacerbated this situation; there was a 31% increase in overdose deaths (including those attributed to synthetic opioids and stimulants, such as cocaine and methamphetamine) from 2019 to 2020 [2] and a further 14% in 2021 [3, 4], with deaths exceeding 100,000 in 2021 and 2022 [5]. The primary driver of opioid overdose deaths in the U.S. is illicitly manufactured fentanyl [6], a synthetic opioid approximately 50 times more potent than heroin [7]. Recent data point to a drug supply that is largely contaminated with fentanyl [8]. In the absence of drug checking, people who use drugs are at increased risk for drug poisoning, which can happen intentionally or unintentionally when drugs are unknowingly adulterated [9, 10]. Although some people who use drugs are aware they are using fentanyl, a large proportion of the overdose burden is attributed to unintentional use, as many illicit substances, including both opioids and stimulants, are adulterated with fentanyl of unknown concentration [9, 10]. Recent research has documented a growing number of people who use more than one drug (e.g., polysubstance use) experiencing overdose [11].

There have historically been variations in overdose mortality rates and fentanyl proliferation across US regions. In 2016, states with the highest opioid death rates included West Virginia, New Hampshire, Ohio, Maryland, and Massachusetts [12]. The populations and regions most impacted by fatal overdose and the types of drugs involved have changed over time. For example, between 2016 and2018, white populations experienced a statistically significant decrease in overdose deaths while overdose deaths began to increase among Black populations [13]. Similar increases in fatal overdoses have been occurring in Hispanic populations [14]. Fatal overdose death rates were also higher in areas with greater income inequality and a larger proportion of Black residents [15].

Between 2015 and 2016, Eastern states had the largest increase in synthetic opioid overdose deaths, while the Midwest saw the largest increase in psychostimulant-involved overdose deaths. Then, from 2018 through 2019, synthetic opioid overdose deaths increased in the west, while psychostimulant-involved overdose deaths increased in the Northeast [16]. Finally, the COVID-19 pandemic has exacerbated overdose mortality overall, resulting in sharp increases of fatal overdoses, widening social and economic disparities, and decreased access to resources [17–20].

Given the extensive literature on overdose prevention in urban areas with greater access to resources including harm reduction, it is important to understand whether and how the measures/strategies people who use drugs in geographically diverse rural settings take to protect themselves. Health-related practices that urban people who use drugs enact to embed safety and risk reduction in their networks has long been an endogenous practice [21–27]. Understanding these behaviors in rural settings could inform future interventions to reduce fatal overdoses. Past research in mostly urban settings has highlighted a variety of strategies that people who use drugs employ to mitigate overdose risks. These include buying drugs from trusted sellers [28]; sniffing, snorting, or smoking rather than injecting [29]; using drugs in smaller increments; and testing the potency of their drugs by injecting a “test dose” [30]. There are also tools available to reduce the risk of overdose and to reverse opioid-related overdoses. Fentanyl test strips can be used to test drugs prior to use [31, 32], and although they do not provide information about how much fentanyl is present, drug-using behaviors may be altered as a result [33]. More advanced drug checking techniques, such as spectrometry, can identify contaminants in the drug supply, but these are rare in the U.S. and often cannot determine sample concentrations [34].

In the event of an overdose, naloxone, an opioid antagonist, can be used to reverse it, although it is not always available and even when it is, barriers such as stigma and fear of arrest discourage carrying it [35]. Most prior studies have focused on urban populations and those conducted in rural areas have had modest sample sizes. Given limited resources and barriers to harm reduction tools and strategies in rural communities, this study sought to explore a large, regionally diverse U.S. sample of rural people who use drugs and the strategies they employ to prevent fatal overdose.

### Rurality in the United States

People who use drugs in rural settings often have reduced access to resources compared to their urban counterparts increasing the risk for overdose fatality. For example, rural populations are more likely to be under- or uninsured and have difficulty accessing healthcare, due, in part, to transportation obstacles [36–40] and fewer physicians practicing in rural areas compared to urban areas [40]. Rural communities also often lack providers of medications for opioid use disorder (MOUD) and harm reduction services including sterile syringe exchange and access to naloxone, both of which are associated with decreased overdose risk [41, 42].

Research has also identified structural and community factors specific to rural populations. For example, stigma can be high in rural settings for people who use drugs [43–47], and there may be fewer economic opportunities (Walzer, 2003) that, in turn, increase stress and other psychosocial issues associated with drug use [48, 49]. Together, lack of access to services, stigma, and economic deprivation create unique overdose risk environments in rural settings [50]. This investigation sought to explore ways in which rural people who use drugs employed harm reduction techniques to prevent fatal overdose.

### Harm reduction as a theoretical framework

Harm reduction is a pragmatic and compassionate public health approach that focuses on minimizing the adverse health, social, and legal impacts associated with drug use, rather than solely aiming to eliminate drug use itself [51]. Central to harm reduction theory is the acknowledgment of the complex and multifaceted nature of drug use, recognizing that it stems from a variety of factors including social, economic, and psychological influences. This approach acknowledges complexities and seeks to improve individual and community well-being by providing strategies and services that reduce risks. In this paper we use harm reduction as a theoretical basis for examining how people who use drugs mitigate the risks of fentanyl exposure and fatal overdose.

Policy responses to drug use have historically been framed by the notion that it is an inherently negative activity best approached by promoting complete abstinence. Harm reduction as a policy for addressing infectious diseases associated with illicit drug use, emerged in the 1980’s primarily in the United Kingdom, Australia and Netherlands. This approach views drug use through a value-neutral lens and argues that minimizing drug use harms should be the primary aim of policy responses [52–55]. Harm reduction does not stigmatize individuals who use drugs, rather it advocates for policies and interventions aimed at reducing the harms, many of which (e.g., policies) are socially constructed [53, 56, 57].

Harm reduction interventions are grounded in evidence-based practices that have been shown to decrease the spread of infectious diseases, prevent overdose deaths, and engage people who use drugs in treatment and care without requiring abstinence. Some examples include syringe services programs (SSPs), drug testing services, and safe consumption sites (also termed overdose prevention centers). SSPs have been studied extensively and are associated with substantially reduced rates of HIV and HCV transmission as well as reduced rates of both fatal and non-fatal overdose [56–59]. Similarly drug testing and overdose prevention centers are associated with reduced fatal overdoses [60–63]. Harm reduction perspectives conceptualize the harms of substance use within their larger social context (i.e., criminalization of drug use and/or drug paraphernalia laws) rather than solely on the individual level [55, 64–66]. Structural factors are thought to constrain and stigmatize activities involved in drug use and harm reduction behaviors in ways that cause harm [67].

Given that many formal harm reduction interventions are inaccessible to people who use drugs, especially those in rural settings [68, 69], this study explores actions that people individually and collectively take to reduce the risk of fatal overdose. There are limited prior studies that highlight protective behaviors among people who use drugs. Past qualitative studies in the U.S. (mostly conducted in urban areas) have found that people alter their drug use by ingesting less or changing the route of administration to reduce the risk of fatal overdose [30]. Other studies, one in Rhode Island study [28] and one in rural Southern Illinois [70] found an increased reliance on drug sellers to inform them about contaminants in the drug supply and felt more secure purchasing from people they trusted. Studies have also explored naloxone access and use [35, 71], noting the concern that naloxone is often not available in rural areas [72]. Considering the ongoing elevated risks, it is critical to document all of the strategies—both evidence-based and otherwise – utilized by people who use drugs to prevent overdose in rural settings.

## Methods

### Study background

This analysis used qualitative data from the Rural Opioid Initiative (ROI) for this study. The ROI was developed in 2015 when the US was experiencing high levels of opioid use and injection drug use that disproportionately impacted rural communities [73]. Over the course of the study, recognition of the increasingly significant role of polysubstance related drug overdose deaths has increased and recognition that the US is currently passing through a wave of the overdose that is characterized by mortality related to polysubstance use. The ROI was funded by the National Institute on Drug Abuse (NIDA), in partnership with the Appalachian Regional Commission (ARC), the Centers for Disease Control and Prevention (CDC) and the Substance Abuse and Mental Health Services Administration (SAMHSA), and consisted of eight research sites located in rural areas. All sites aimed to understand drug use in their local area and to develop comprehensive approaches to prevent and treat harmful outcomes related to drug use. NIDA also funded the Rural Comorbidity and HIV Consequences of Opioid Use Research and Treatment Initiative Data Coordinating Center (DCC) that compiled data from all sites, including qualitative data.

Rurality was defined based on the US Health Resources and Services Administration (HRSA) “Am I Rural” website. The website uses definitions of rurality from several US federal programs, including the US Census Bureau, the Office of Management and Budget (OMB), and the Economic Research Service of the U.S. Department of Agriculture (USDA-ERS). The federal ROI funding agencies used this website to inform research study selection and study sites confirmed rurality and other indicators of vulnerability during the application process.

The eight research sites spanned 65 counties in 10 states (Illinois, Kentucky, Massachusetts, New Hampshire, North Carolina, Ohio, Oregon, Vermont, West Virginia, and Wisconsin) with high rates of substance use, overdose, and infectious disease rates [73]. Given the large geographic span, there was diversity not only in location but also in participants and local drug use practiced and norms. For example, New England (NE) (referred to as the NE study site throughout this paper) included contiguous communities located along the Connecticut River Valley in western Massachusetts, Eastern Vermont, and western New Hampshire. Some sites were located in Appalachia, a cultural and geographical region that spans 13 states from New York to Mississippi. These included Eastern Kentucky, western North Carolina, West Virginia, and Southeastern Ohio. All rural communities included in the study have experienced increased vulnerability to fatal overdose, HCV transmission and concern for HIV transmission due to high rates of injection drug use and limited access to healthcare [74–76].

The first phase of the ROI included epidemiologic and policy scans, and the collection of harmonized qualitative and quantitative data. The second phase focused on site-specific and data-informed interventions, which differed by site depending on the needs identified in the initial phase. This paper uses qualitative data from the first phase to describe harm reduction techniques that participants employed to reduce their risk of experiencing fatal overdoses.

Rurality was defined using the US Health Resources and Services Administration (HRSA) “Am I Rural” website. The website uses definitions of rurality from a number of US federal programs, including the US Census Bureau, the Office of Management and Budget (OMB), and the Economic Research Service of the U.S. Department of Agriculture (USDA-ERS). ROI funding agencies used this website to inform site selection and study sites confirmed rurality and other indicators of vulnerability during the application process.

The eight research sites spanned 65 counties in 10 states (Illinois, Kentucky, Massachusetts, New Hampshire, North Carolina, Ohio, Oregon, Vermont, West Virginia, and Wisconsin) with high substance use, overdose, and infectious disease rates (see https://ruralopioidinitiative.org/studies.html for more information about each site) [73]. Given the large span of rural counties, there was diversity in location, participants, and drug use norms. New England (NE) (referred to as the NE study site throughout this paper) included contiguous communities located along the Connecticut River Valley in western Massachusetts, Eastern Vermont, and western New Hampshire. Some sites were located in Appalachia, a cultural and geographical region that spans 13 states from New York to Mississippi. These included Eastern Kentucky, western North Carolina, west Virginia, and Southeastern Ohio. Wisconsin and Illinois sites were located in rural Midwestern US counties. Oregon included coastal and land-locked communities in large, thinly populated counties. All rural communities included in the study have experienced increased vulnerability to fatal overdose, HCV transmission and concern for HIV transmission due to high rates of injection drug use and limited access to healthcare [74–76].

The first phase of the ROI involved epidemiologic and policy scans, and the collection of harmonized qualitative and quantitative data. The second phase of the ROI focused on data-informed and site-specific interventions, which differed by sight, depending on the identified needs in the first phase. This paper uses qualitative data from the first phase to describe qualitative findings focused on harm reduction techniques rural participants employed to reduce their risks of experiencing fatal overdoses.

### Semi-structured qualitative interview guide

A cross-site ROI working group with expertise in qualitative methods developed and harmonized the interview guide used across sites. Interview domains included: background (e.g., specifics to the region such as questions tailored to the local drug market, social networks, family); past and current drug use; drug use behaviors; overdose experiences; sexual behaviors; interactions with law enforcement; and experiences with healthcare and other service providers. Specific questions about fentanyl were indicated as probes. People who indicated that they injected drugs could be asked: “*Have you ever used any drugs containing fentanyl?”, “Did you realize before or after you took the drug that it contained fentanyl?”, and “Were you seeking a drug that contained fentanyl, or were you unaware?”* Outside of these questions, fentanyl discussions emerged from participant narratives, often when they were describing overdose experiences. Although the fentanyl probes were listed under the question about injection drug use, the interviews were semi-structured and allowed for the interviewer to probe at any time during interviews. See Online Appendix A for the interview guide.

There were no harmonized questions about fentanyl test strips, which were not in wide use when the study was designed; thus, this information emerged through the semi-structured methodology, which allowed participants to talk freely and for interviewers to probe. Similarly, there were no harmonized questions about naloxone; however, questions in the interview guides were used as probes to the following question: *“Now I’ll ask about your experience with overdosing, which includes if you passed out, turned blue, or stopped breathing from using drugs. Have you ever overdosed?”* If participants answered yes to overdosing, they were asked, *“Tell me about the most recent time that you overdosed.”* Probes to this question, which could have been asked in the interviews, included: *“Was Narcan/naloxone used? If yes, who administered the Narcan/naloxone first?”* Similarly, participants were asked about witnessing someone else overdosing. If participants responded yes, the same probes were used. to witnessing an overdose, they could receive the following probes: “*Was Narcan/naloxone used?”* Participants who responded yes could also be asked:*“Do you currently have Narcan/naloxone with you or at home?”* and* “If you wanted to get Narcan/naloxone, do you know how to get it?”.*

Each study received approval from the local institutional review board and participant privacy was protected by a federal Certificate of Confidentiality.

### Participant recruitment and data collection

Between 2018 and early 2020, people who use drugs were recruited to participate in a 60–90-min semi-structured qualitative interview. Eligibility criteria and recruitment methods differed slightly by site (Table 1); however, all participants resided in the study area and disclosed recent opioid use or injection drug use. Participation of individuals who may not have used opioids but did inject drugs was more common in areas where methamphetamine use was prominent. All sites recruited participants from community-based programs, and in some cases used novel community-based recruitment methods, such as community cookouts [77]. Study staff trained in qualitative methods conducted interviews, which were digitally recorded and transcribed. All participants gave written informed consent to participate and were compensated between $25–50 depending on local site protocol.

**Table 1:** Eligibility criteria and qualitative recruitment method, by site

Site	Eligibility criteria	Qualitative recruitment method
IL	Age ≥ 15 years	Participation in the study site questionnaire/UG3/ ROI survey (i.e., participation in the quantitative study)
	Current resident in study area	
	English-speaking	
	Opioid use or methamphetamine injection in past 30 days	
	Accepts referral to harm reduction services	
KY	Age ≥ 18 years	Participation in the study site questionnaire/UG3/ ROI survey (i.e., participation in the quantitative study)
	Current resident in study area	
	English-speaking	
	Injected drugs or used opioids to get high within past 30 days	
NC	Age ≥ 18 years	Participation in the study site questionnaire/UG3/ ROI survey (i.e., participation in the quantitative study)
	Current resident in study area and intends to stay 12 months	
	English-speaking	
	Opioid or methamphetamine injection in past 30 days	
	Verification by stigmata or appropriate description of injection practices	
	Indication in questionnaire of having injected painkillers or heroin	
NE	Age ≥ 18 years	Convenience sample of in-depth interview participants through street outreach, venue-based recruitment, and respondent driven sampling from the UG3/ROI survey (i.e., participation in the quantitative study)
	Spent most of the last 30 days in the study area	
	Used opioids to get high or injected any drug in the last 30 days	
	Able to provide informed consent	
	English-speaking	
OH	Age ≥ 18	Community partners and key informants
	Current resident in study area	
	Used heroin or prescription opioids or injected any type of drug to get high in the past 30 days	
OR	Age ≥ 18	Advertisement in community-based and service locations; direct recruitment by service provider and outreach staff
	Current resident in study area	
	English-speaking	
	Injected any drug or used opioids to get high in the past 30 days	
WI	Age ≥ 15	Participation in the study site questionnaire/UG3/ ROI survey (i.e., participation in the quantitative study)
	Current resident in study area	
	Injected any opioid drug in the past 30 days	
WV	Age ≥ 18	Direct recruitment by outreach staff and service providers of dynamic individuals’ well-known communities of people who use drugs
	Current resident in study area	
	Injection drug us in past 30 days	

United States abbreviations are as follows: Illinois (IL), Kentucky (KY), North Carolina (NC), New England (NE), Ohio (OH), Oregon (OR), Wisconsin (WI), West Virginia (WV)

Recruitment and data collection also were context specific. For example, some sites' local drug markets were dominated by methamphetamines, and therefore, to understand the specific contexts and risks for overdose, those sites proactively recruited people who used methamphetamines. Although each ROI study site had to adapt to the specific needs of their community, the study sites remained in contact and when possible aligned methods, harmonized the qualitative interview guide, and worked collaboratively across sites throughout data analyses. More information about the ROI has been published elsewhere [73].

### Data analysis

Researchers with experience in anthropology, epidemiology, public health, addiction medicine, harm reduction, and social services conducted in-person interviews at each site. Interviews were audio recorded for all sites and professionally transcribed. All transcripts were de-identified and assigned a unique identification number, then uploaded to a qualitative software program (Dedoose, Los Angeles, CA, v. 09.0.62) for data management, coding, and analyses. Researchers with expertise in qualitative methods from the ROI Data Coordinating Center (DCC) conducted preliminary coding to categorize data by interview topic areas and lines of inquiry. This preliminary coding used a deductive coding technique that aligned codes to the interview questions. The DCC created a code book that included all ROI site data.

We then comprised the writing team with representatives from all ROI sites to analyze data compiled by the DCC. The interdisciplinary writing team had expertise in epidemiology (TS, AR, MF), anthropology (RF), sociology (SW, DF, RB, LO) public health (WZ, TS, SW, RB, AS, VG, LO, MP, DS, JF, DS, GS) infectious disease (MP, DS, JF), and drug use (WZ, TS, SW, RB, AS, VG, LO, MP, DS, JF, DS, GS). We explored all codes that were related to overdose and fentanyl and used an inductive thematic analysis approach to identify emergent themes and create new codes accordingly. This is a data-driven coding method that has similarities to grounded theory [78] in that the data dictates the codes; however, it is done after data collection [79]. During monthly meetings team members presented the themes that emerged from the data they had reviewed. To ensure intercoder reliability, three people were assigned to evaluate initial coding. All coding decisions were discussed in our writing team meetings until a consensus was reached.

During this inductive coding process, the study team identified harm reduction techniques that study participants referenced in their narratives about fentanyl. We consolidated the harm reduction themes as needed and created appropriate sub-codes. Since the writing team only explored data that had been deductively coded in the initial code book, developed by the DCC, which focused on overdose and fentanyl, we queried the entire dataset for specific words that emerged from our inductive coding that could potentially have been missed in the DCC deductive coding scheme. After including additional quotes, we finalized the codebook, reviewing the codes to identify redundancies and potential gaps and refining code definitions to ensure accuracy and clarity.

## Results

A total of 349 qualitative interviews were included in this analysis. Slightly over half of participants were male, with ages ranging from 20 to 63 years old, and most participants reported being white. Table 2 provides demographic information by site.

**Table 2:** Demographic characteristics of 349 people who used drugs in 8 rural US studies, 2018–2020

	Total	Illinois	Kentucky	North Carolina	New England	Ohio	Oregon	Wisconsin	West Virginia^f^
Interviewees (data sent by studies used)	349 (100%)	22 (6%)	57 (16%)	65 (19%)	22 (6%)	26 (7%)	52 (15%)	60 (17%)	45 (13%)
Male^a^	194 (64%)	14 (64%)	35 (61%)	34 (52%)	10 (45%)	15 (58%)	28 (54%)	33 (55%)	25 (56%)
Average age	36	37	35	36	33	37	39	35	38
Race									
White	213 (61%)	20 (91%)	56 (98%)	15 (23%)	15 (68%)	*not asked*	49 (94%)	58 (97%)	(0%)
Black	2 (1%)	2 (9%)	0 (0%)	0 (0%)	0 (0%)	–	0 (0%)	0 (0%)	0 (0%)
Native American	8 (2%)	0 (0%)	0 (0%)	5 (8%)	1 (5%)	–	1 (2%)	1 (2%)	0 (0%)
Mixed race	5 (1%)	0 (0%)	0 (0%)	2 (3%)	0 (0%)	–	2 (4%)	1 (2%)	0 (0%)
Other	1 (0%)	0 (0%)	1 (2%)	0 (0%)	0 (0%)	–	0 (0%)	0 (0%)	0 (0%)
Not given/Not asked^b^	120 (34%)	0 (0%)	0 (0%)	43 (66%)	6 (27%)	26^2^ (100%)	0 (0%)	0 (0%)	45 (100%)
Interviewees (coded interview data used)	349 (100%)	22 (6%)	57 (16%)	65 (19%)	22 (6%)	26 (7%)	52 (15%)	60 (17%)	45 (13%)
Substance use									
Ever IDU	331 (95%)	20 (91%)	57 (100%)	65 (100%)	21 (95%)	17 (65%)	52 (100%)	56 (93%)	43 (96%)
Current IDU	318 (91%)	19 (86%)	57 (100%)	65 (100%)	18 (82%)	15 (58%)	50 (96%)	55 (92%)	39 (87%)
*Heroin*	214 (61%)	8 (36%)	30 (53%)	51 (78%)	18 (82%)	13 (50%)	41 (79%)	34 (57%)	19 (42%)
*Fentanyl*	78 (22%)	1 (5%)	17 (30%)	26 (40%)	14 (64%)	3 (12%)	6 (12%)	6 (10%)	5 (11%)
*Methamphetamine*	225 (64%)	14 (64%)	37 (65%)	61 (94%)	4 (18%)	6 (23%)	47 (90%)	41 (68%)	15 (33%)
*Other*	226 (65%)	4 (18%)	54 (95%)	61 (94%)	15 (68%)	10 (38%)	21 (40%)	28 (47%)	33 (73%)
Surveyed Interviewees^c^ (survey data used)	144 (100%)	22 (15%)	23 (16%)	23 (16%)	16 (11%)	NA	NA	60 (42%)	NA
Substance use									
Current (Past 30 days)	143 (100%)	22 (100%)	23 (100%)	23 (100%)	16 (100%)	NA	NA	59 (98%)	NA
Recruited by									
Service or program staff	6 (4%)	2 (9%)	0 (0%)	4 (17%)	0 (0%)	NA	NA	0 (0%)	NA
Other	83 (58%)	11 (50%)	14 (61%)	9 (39%)	14 (88%)	NA	NA	35 (58%)	NA
Not indicated	55 (38%)	9 (41%)	9 (39%)	10 (43%)	2 (13%)	NA	NA	25 (42%)	NA
Interviewees (data sent by studies used)	304 (100%)	22 (7%)	57 (19%)	65 (21%)	22 (7%)	26 (9%)	52 (17%)	60 (20%)	NA
Education									
< High school	56 (18%)	1 (5%)	21 (37%)	13 (20%)	3 (14%)	7 (27%)	*not asked*	11 (18%)	*not asked*
H.S. or GED	97 (32%)	11 (50%)	22 (39%)	21 (32%)	9 (41%)	11 (42%)	–	23 (38%)	–
Some college	61 (20%)	7 (32%)	6 (11%)	20 (31%)	3 (14%)	6 (23%)	–	19 (32%)	–
Assoc/trade deg	16 (5%)	3 (14%)	8 (14%)	0 (0%)	1 (5%)	0 (0%)	–	4 (7%)	–
> = B.A	12 (4%)	0 (0%)	0 (0%)	8 (12%)	0 (0%)	1 (4%)	–	3 (5%)	–
Not answered^d^	62 (20%)	0 (0%)	0 (0%)	3 (5%)	6 (27%)	1 (4%)	52^d^ (100%)	0 (0%)	–
Income Source									
Full-time work (40 h/wk)	44 (14%)	*not asked*	6 (11%)	13 (20%)	0 (0%)	10 (38%)	*not asked*	15 (25%)	*not asked*
Part-time work	26 (9%)	–	6 (11%)	7 (11%)	2 (9%)	1 (4%)	–	10 (17%)	–
Retirement check	2 (1%)	–	2 (4%)	0 (0%)	0 (0%)	0 (0%)	–	0 (0%)	–
Public assistance check – like TANF, AFDC etc	29 (10%)	–	29 (51%)	0 (0%)	0 (0%)	0 (0%)	–	0 (0%)	–
Disability check, like SSI, military or other	19 (6%)	–	0 (0%)	10 (15%)	4 (18%)	1 (4%)	–	4 (7%)	–
Selling drugs	4 (1%)	–	0 (0%)	0 (0%)	1 (5%)	0 (0%)	–	3 (5%)	–
Selling sex	1 (0%)	–	0 (0%)	0 (0%)	0 (0%)	0 (0%)	–	1 (2%)	–
Theft, shoplifting etc	3 (1%)	–	0 (0%)	0 (0%)	1 (5%)	0 (0%)	–	2 (3%)	–
Someone supports me	15 (5%)	–	0 (0%)	0 (0%)	2 (9%)	0 (0%)	–	13 (22%)	–
Other	12 (4%)	–	0 (0%)	0 (0%)	6 (27%)	1 (4%)	–	5 (8%)	–
Unemployed	59 (19%)	–	13 (23%)	34 (52%)	0 (0%)	12 (46%)	–	0 (0%)	–
Missing^e^	90 (30%)	22^e^ (100%)	1 (2%)	1 (2%)	6 (27%)	1 (4%)	52^e^ (100%)	7 (12%)	–

^a^One interviewee from OR identified as “Neither”male of female

^b^Some NC and NE interviewees did not give their race/ethnicity. OH did not ask interviewees their race/ethnicity

^c^Some, but not all, participants completed a quantitative survey before their interview

^d^OR did not ask interviewees about education

^e^IL and OR did not ask interviewees about income source. WI had 4 “Refuse to answer and 3 “Don’t know” responses. KY, NC, NE and OH had interviewees who did not answer the question

^f^WV did not ask questions about race, ethnicity, or education

^g^Ethnicity data (e.g., Hispanic, non-Hispanic) was not collected

^h^United States abbreviations are as follows: Illinois (IL), Kentucky (KY), North Carolina (NC), New England (NE, including Massachusetts, New Hampshire, and Vermont), Ohio (OH), Oregon (OR), Wisconsin (WI), West Virginia (WV)

### Fentanyl awareness

Participants were aware of fentanyl contamination in their drug supply and noted that its presence was common whether they used opioids or stimulants. Participants explained: *“Every other bag of heroin you get, or every other fake 30 you get, is cut with fentanyl”* (West Virginia, male, age 20). Another participant said, “*You gotta watch ‘cus some of the uh, the ice [methamphetamine] you get is cut with fentanyl and it’ll kill you”* (Ohio, male, age 43).

Despite the awareness that the drug supply likely contained fentanyl, most participants expressed some uncertainty regarding their own unintentional use. Participants described overdosing and shared that they suspected fentanyl was in the drugs they used because the effect was stronger than normal. Participants said things like, *“I think I did fentanyl. Somebody told me it was China White [heroin], but I’m pretty sure it was fentanyl”* (Oregon, male, age 25). When describing his most recent overdose one participant said,

“It was either China or fentanyl. I was thinking it was fentanyl because it was so strong. Just a little bitty speck would put you in the floor almost. And I had probably done close to a tenth of a gram and mixed with a little meth. And I guess I fell out on the side of the wall into the toilet, against the wall. So, I'm leaned over against the wall, and I was passed out there. And they found me, and they woke me up, and my old lady tripped out on me, and she left” (Kentucky, male, age 31).

Participants who use stimulants were also concerned about fentanyl adulteration in their drug supply. When discussing her methamphetamine use, one participant said,

“There’s been a couple times where like me and whoever I’d be around- were like well, that must’ve been mixed with fentanyl because we did it and we would just go right to sleep, just nod off, and I felt like I was on heroin for a couple of times, and it was like weird” (Wisconsin, female, age 34).

Experiences such as this led to concerns about the quality of drugs available, especially methamphetamine, because it seemed that fentanyl contamination had occurred more recently than in the heroin supply, and therefore was less expected.

“My thought about fentanyl in general is it should not [be] able to be around, and I don’t know how they’re doing it but that’s why everybody’s getting so fucked up. You know you go and shoot meth and you’re not shooting just meth, it’s fentanyl, and it’s fucking people up” (Wisconsin, female, age 34).

One man confirmed these suspicions through a positive drug screen: *“I do the meth, the crystal, or the ice what they call it now. And even at that, that stuff is laced with all kinds of stuff. When I was at the Suboxone clinic they pulled blood, and they said I had fentanyl in me. And I hadn't done fentanyl in years, and it had to have come from the ice”* (Kentucky, male, age 61).

Despite regional trends in drug use, we did not see regional differences in fentanyl awareness. This is likely because participants, regardless of location, reported the perception that their drug supply was contaminated with fentanyl. Given the absence of a regulated drug supply and the ability to check drugs prior to use, participants developed several strategies to avoid overdoses associated with fentanyl. Most used multiple strategies at once to ensure success and avoid fatal overdose. Sometimes decisions were affected by whether participants were using heroin or methamphetamine, with many participants expressing greater concerns over adulteration when using heroin.

### Harm reduction strategies

#### Avoiding heroin

Participants described using a variety of strategies to avoid unknowingly ingesting fentanyl; in particular, avoiding heroin was one theme that emerged. “*I said “No more heroin. I ain’t using any more heroin. I don’t want to lose my life…Pain pills maybe, but no more heroin and no fentanyl, either*” (Oregon, female, age 60). Other people talked about experiences with people close to them overdosing on heroin with fentanyl, which prompted their concerns and decision to avoid heroin:

“I’ve had friends overdose on heroin, and I’ve had a couple die. That is what probably deterred me from ever using heroin. You know, like, you have some of the fentanyl, and what they put in it for cut, and you see it on TV and all the overdose, the deaths, and they talk about it continuously” (Wisconsin, male, age 47).

A woman in Oregon said:

“See, the heroin was getting cut with fentanyl for a while there in [location redacted]. I’m pretty sure I tried it, but I’ve seen my really good friend, and he almost died. Another friend of mine did die and they had to bring her back, and then my baby cousin died. So, I was not touching it. I’m not touching it definitely with a needle” (Oregon, female, age 39).

The above participant says she will not use heroin, but at the end of her sentence says “definitely not with a needle” suggesting there may be a time she will use heroin, but she will exercise caution and change the way she uses it to protect herself.

#### Buying from trusted people who sell drugs

Since there was a palpable awareness and concern about potential fentanyl contamination in all drugs including stimulants, participants employed additional harm reduction measures. One tactic used was buying drugs from a trusted person. This is because *“There’s some dealers that you trust more than others”* (New England, male, age 39). Other participants described:

“I absolutely will not buy from somebody that is a stranger ... because strangers don’t care. Um, I got to know who they are, and they have to know who I am, and have some degree of you know, respect between us before I’ll buy anything” (Ohio, female, age 32).

Many participants described their relationships with the people from whom they purchased drugs as being built on trust and friendship. There was genuine care for each other and the people who sold drugs would look out for their customers: “*My guy, specifically, he’s like “I don’t think I’m gonna sell it to you, man, because I don’t want you dying on me. And he actually did have concern for me, you know, he was still selling me heroin but he didn’t want me to die.”* (Wisconsin, male, age 29). Stated another person:

“First time I did fentanyl-based heroin, which is more fentanyl than heroin, dude was straight up with me that sold it to me, he's a daily user, daily shooter and he said, "I'm going to give you this shit. I'm going to say, you have to swear to me that you will not shoot it." And I didn't have to shoot at the time anyway, so you didn't have to worry about me doing that” (North Carolina, female, age 28).

These trusted relationships that were built between participants and drug sellers served as protective in some cases, as participants would alter their drug use behaviors depending on what information was shared with them about the drugs they were selling, as the participant below explains.

“I remember my dealer once got bags of fentanyl, and they knew it was fentanyl, and they told me just do one, and I did” (New England, male, age 29).

#### Altering drug use practices

Given the growing concern about fentanyl and many personal experiences with overdose, participants altered their drug use practices. This included avoiding injecting; using smaller amounts; tasting, smelling and/or examining drugs, and using drugs with other people.

#### Avoiding injecting

When participants were unsure about their drugs, but still wanted to use, they described doing so in ways that reduced the risk of fatal overdose. This often meant avoiding injecting.

Said one woman, “*If I'm concerned about it, if I'm told that it's just pure fentanyl-based, I'm not shooting [injecting] it”* (North Carolina, female, age 28).

Some participants recounted testing heroin by smoking it first and then making a decision to continue using the heroin by smoking or injection: “*We smoked the heroin, any heroin we’ve got you smoke a little bit. Just inhale it, take a score and see what it is, ‘cause if you’re going to- I mean you can smoke the fentanyl*” (Wisconsin, male, age 27).

#### Testing small amounts of the drug

Participants also discussed first using smaller amounts via injection.

“Now whenever I use heroin, like if I'm with some people and we get a new batch, I'll guinea pig it because other people will do just ... they'll do a shot [inject] like they would ... like this dope was the same dope you had last week. Guess what? It's not, jackass. It's not. I would do a tenth of a tenth, a very small amount. Ten units of water. That's all I needed. Very small amount to gauge the potency. I'm pretty good at being able to tell. "Okay, this is ... whoa, be careful, guys. That's all I can tell you. Be very careful" (Illinois, male, age 25).

#### Tasting, smelling, and/or examining drugs

Participants also discussed using sensory methods to investigate their drugs. As described by one person, “*I mean, usually most of the fentanyl-based heroin is going to be lighter in color. Most of the heroin here is brown”* (North Carolina, female, age 43). Another woman said:

“Straight heroin taste is a more vinegary flavor. The fentanyl doesn’t have that flavor. It doesn’t have the vinegary. You can also take a black light. If you have heroin, you can take a black light and it will florescent-green. It will florescent-green if it’s heroin” (Oregon, female, age 50-59).

North Carolina had an important regional theme emerge where people discussed a type of heroin referred to as “Gray Death” that was potentially contaminated with fentanyl: *“It was gravel. It was gray, it was gravel gray. I'm sure it had something in it. I didn't have a test strip, but I'm sure it had”* (North Carolina, female, age 28). This was the only location that discussed gray heroin and should be explored more in future research.

#### Using drugs with other people

Another important strategy was using drugs with others so if someone overdosed help could be called and/or naloxone could be used and the overdose could be reversed.

*“When we did it the other day, the girl who brought it over, I watched her do it, saw how much she did. I just watched her and kept my eye on her for a few minutes afterwards to see how she was because I know with the stuff we were doing before, almost everybody would pretty much almost go out, just hardly even keep their eyes open. She just kept talking like a regular conversation, so it wasn't to a point where she couldn't continue to talk. Then my husband almost always does it first. I don't really know why we do that, but we just do”* (North Carolina, female, age 49).

#### Fentanyl test strips

Fentanyl test strips can be used to detect the presence of fentanyl in drugs but they cannot quantify it [33]. Not all participants knew about or had access to fentanyl test strips: *“I have overdosed and it wasn't any more than I'm used to using, so I'm pretty positive that I've absolutely used fentanyl at some point in time. But I know now, they have those little fentanyl test strips and things like that. Getting access to them is virtually impossible*” (North Carolina, male, age 38).

However, another participant in North Carolina reported having access to fentanyl test strips.They explained how they would use them when they felt uncertain about their drugs: *“I use test strips, I try to use test strips when I can”* (North Carolina, female, age 28). This suggests that some participants in North Carolina had greater access to fentanyl test strips than others. More research should be conducted to understand who has increased access and why.

Participants who obtained strips often got them from their local harm reduction organization. For example, a New England participant who was talking about familial drug use described how her older sister used fentanyl test strips and new injecting equipment from the syringe services program:

“She gets, um, the fentanyl testers…she’s very like, she gets high, don’t get me wrong. But she’s very aware of what she’s getting high with. She’s not one of them that you’re just going to give her a bag and she’s going to do it. She’s like a scientist” (New England, female, age 38).

#### Naloxone

Awareness of and access to naloxone was described at most sites, although this varied. Participants in North Carolina exhibited the greatest awareness and access, and described carrying and using Narcan, a name brand of naloxone: “*I always carry one in my purse and then I always have two in my car. Then we have some at home too*” (North Carolina, female, age 25).

Ensuring avoidance of a fatal overdose through the use of naloxone was well articulated among participants and their peers. Stated one woman, “*A lot of people that got it [naloxone] have gone to [Agency] and gotten the kit, the Narcan kits”* (New England, female, age 31). Another person said:

“Our dealer who gave it to us said, ‘I’m not even giving this to you unless you have Narcan because I know it’s strong.’ And we had it on us and I went and then within the same hour my boyfriend’s brother went. And it took two shots for me and five for him. That was a scary day, but we both came back” (Wisconsin, female, age 28).

However, some participants noted that the presence of naloxone was less common in their rural area compared to urban settings:

“Narcan is more common in bigger cities. In Champagne, everyone else at meetings, half of them were trained and had it on them. I used to. Down here, no. There's not a lot of a Narcan presence down here that I'm aware of personally. I could be wrong” (Illinois, male, age 27).

These findings gesture to potential regional differences in naloxone availability or access at the time of this study. This could be due to harm reduction activism and infrastructures in each location.

## Discussion

Participants in this study living in rural regions of the U.S.– regardless of location or type of drug used– described an awareness and growing concern that their drug supply was contaminated with fentanyl. They described worries about fentanyl in opioids, including counterfeit pills, as well as in stimulants, particularly methamphetamines, without their knowledge. Research has identified fentanyl in stimulants as a concern and risk for overdose, now termed “the fourth wave” of the overdose epidemic to be an increase in polydrug use that includes stimulant and opioid use [80–82]. This study adds context to that literature by describing the concerns and uncertainty of a large rural population who use drugs [83, 84]. Prior studies have found that rural areas are experiencing growing rates of methamphetamine use, both alone and in combination with other substances [85] and 74% of survey respondents in the quantitative ROI study reported methamphetamine use in the past 30 days [73]. This finding highlights the need for targeted overdose prevention approaches for people who use stimulants and those who use multiple drugs. Those with concerns about fentanyl-contaminated methamphetamine took additional measures to protect themselves and others from overdose (Table 3).

**Table 3:** Harm reduction organizations and services, by site 2018–2020

Site	Harm Reduction Services
IL	1 syringe service program (SSP). The SSP had 2 fixed locations and operated a mobile unit that served the 16 southern counties of IL
KY	2 SSPs. Both were fixed locations located in 2 separate counties
	An additional fixed location SSP opened near the end of the study, but most of the recruitment was complete so the results of this study likely do not reflect interactions with that SSP
NC	3 fixed location SSPs, 2 of which made limited deliveries. Fixed locations and deliveries spanned 4 counties
NE	5 fixed location SSPs, 4 of which offered mobile services. In total, the 5 SSPs served 7 out of the 11 counties included in this study site
OH	2 SSPs. Both were fixed locations in 2 different counties
OR	14 agencies provided SSP services. Services spanned 19 counties. There were 18 fixed location sights. One of the sites used a delivery model
WI	13 SSPs covering 20 counties of WI within the study catchment area. One out of the 13 SSPs had a mobile unit that served 8 counties (one county overlapped with a fixed location site)
WV	2 SSPs. One was a fixed location and the other mobile. They served 2 counties

Fixed location SSPs are reported as one county (the county they were located in. Although any participant could access those fixed locations due to transportation and other various barriers it was challenging to go out of one’s county

Participants who used opioids, stimulants, or both engaged in several individual and community-level harm reduction techniques to prevent fatal overdose, often using multiple approaches simultaneously. One common method was purchasing drugs from people they trusted. Participants recounted being warned about the possible presence and danger of fentanyl by trusted sellers who showed genuine concern for the buyers’ wellbeing. Some people who sold drugs even supplied harm reduction tools, such as syringes, fentanyl test strips and naloxone. This finding echoes past research that found people who sell drugs often care about the people they sell to and actively try to prevent overdose or other drug related harms [28, 62]. Findings from this large rural sample add significantly to our understanding of how people who sell drugs interact with their customers. Although sellers may not be aware of all the contaminants in the drugs they sell, both sellers and people who use drugs can alert others that overdoses have occurred from a particular supply or whether the supply tests positive for fentanyl [86, 87].

These findings underscore that persons who sell drugs can, as social network members, be actors in improving health outcomes for people who use drugs [88–90]. Research suggests that laws criminalizing people who sell drugs do not reduce access to drugs, but may push people to buy drugs from unknown sellers, potentially increasing overdose risk [91]. In fact, a recent study found that overdose clusters increased following police drug seizures [92, 93]. When people, including those selling drugs, are removed from their social networks the entire network can suffer and negative health outcomes can occur [94]. Our findings suggest a potential opportunity to look for ways to optimize established social networks of people who use drugs to prevent overdose and disseminate harm reduction strategies. Providing people who sell and use drugs with resources to check their drugs would also be useful, helping both to better understand what is in the local drug supply and discuss drug-checking informed harm reduction approaches, as noted in a recent study in an urban setting [95].

Altering drug use behaviors was another harm reduction strategy described by participants. Participants (1) avoided injecting, (2) tested small amounts of their drug(s) before using, (3) tasted, smelled and/or looked at drugs to identify fentanyl, and (4) used drugs with other people. Using smaller amounts of drugs, often referred to as “test shots”, has been previously documented [96, 97]. People also can change the way they use by switching from injecting to snorting or smoking, which can reduce overdose risk [98]. Research in Canada found that people reported looking for differences in “color, odour, taste” in their drugs to identify fentanyl and, if they were concerned, they would alert friends, but these techniques have proven ineffective in identifying fentanyl contamination [99]. On the other hand, using with other people is an effective harm reduction strategy [100] and was an important technique described by participants in this study since most fatalities occur among people who use drugs alone [101, 102].

Few participants mentioned fentanyl test strips, which can be used to detect the presence of fentanyl in drugs; however, this study was done just as fentanyl test strips were becoming available. This echoes other studies in rural areas that report a lack of awareness about fentanyl test strips [96] and shows that the problem is widespread across rural areas of the U.S. Providing fentanyl test strips to people who use drugs, both stimulants and opioids [103], is an approach to scale up overdose prevention efforts. However, fentanyl test strips only signal the presence of fentanyl and not its potency [33]. At the very least, test strip detection of fentanyl can be used to indicate the need for additional harm reduction measures. Many people who use drugs in rural areas lack access to formal harm reduction services and other health supports [47], making it difficult to obtain tools like fentanyl test strips. One way to increase accessibility to fentanyl test strips is by mailing them to people living in areas that lack access to harm reduction services [104]. Fentanyl test strips could also be distributed in key social spaces, such as libraries, pharmacies, and vending machines in key locations [105]. People who sell drugs could also distribute test strips.

There have been community efforts to provide more sophisticated drug testing than fentanyl test strips alone. For example, the Urban Survivors Union based in Greenville, North Carolina, began using a drug-checking spectrometer to identify contaminants in the drug supply, and in 2022 the University of North Carolina launched a program to check drugs by mail program [34]. Harm reduction agencies in urban locations have also begun to implement drug checking programs [106]. Despite the effectiveness of this type of drug checking{Giulini, 2023 #7593}{Borden, 2022 #7592}, and its wide acceptability internationally, the U.S. has had limited uptake [107]. There has also been an increase in the presence of the veterinary sedative xylazine in drug samples that causes necrotic soft tissue ulcerations [108] and increases the risk of overdose, among other health complications [109, 110], and test strips for this agent are now available. Given that contaminants in the drug supply is an ongoing and evolving problem, access to more sophisticated drug checking is critical.

In regard to naloxone, we identified an important potential regional difference. While North Carolina participants seemed to have more access and experience using naloxone, this regional difference could be related to how questions were asked in the qualitative interview (see qualitative guide in Online Appendix). The quantitative ROI findings reported that 53% of survey respondents ever received “an overdose reversal kit or prescription for naloxone or Narcan” [73] and the New England site reported that 43% of survey respondents had used naloxone to reverse an overdose [111]. Sixty-seven percent of survey respondents in North Carolina reported ever receiving an overdose reversal kit or prescription for naloxone, and while this percentage is higher than the average, it was not the highest percentage reported; in Ohio, 72% of survey respondents reported ever receiving an overdose reversal kit or prescription for naloxone or Narcan [73].

Interestingly, one participant from Wisconsin mentioned receiving naloxone from the person from whom they purchase drugs, suggesting that in the absence of structured harm reduction efforts, people who sell and use drugs may work together to develop strategies to prevent fatal overdose. Of note, naloxone can be costly and access to affordable or free naloxone is critical [112]. Similar to our drug-checking findings, this study highlights the importance of increasing awareness and accessibility to naloxone in rural areas. Two strategies for achieving this aim are collaboration with harm reduction organizations and, in particularly resource-scarce settings, through mail-ordered naloxone.

This study has several limitations. The ROI funded initiative was developed in 2015 and data collected between 2018 and 2020; causes of overdose fatalities have changed over time and the results of the current study reflect the 4th wave and may not reflect the current state, when most overdose fatalities involve multiple substances. The sample is comprised of predominantly white participants which were the majority of residents in the study areas. Future studies should include a more racially and ethnically diverse sample of rural people who use drugs, as their experiences may differ significantly. Additionally, focusing on other characteristics, such as gender, sexuality, and disability, will be important to understand what subpopulations of people who use drugs need to prevent overdose [113]. The increase in people under 30 years of age experiencing overdose suggests that future studies may consider oversampling younger adults [114]. Rural areas within this study are diverse, and variations in geographical, economic, and social factors can lead to significant differences between different rural regions. There were differences in micropolitan areas within some rural regions and more accessibility to micropolitan settings depending on location, which could have impacted access to harm reduction supplies. We do not know if people who sell drugs were in our sample, as we did not ask specific questions about sales experience. Future studies might consider exploring how people who sell drugs use harm reduction methods to prevent overdose for themselves and for their customers. Because interviews were conducted before the COVID-19 pandemic, this study cannot assess COVID’s impact on overdose risk nor harm reduction approaches to prevent overdose. However, recent studies in some of the rural areas represented in this study argue that overdose risk increased after the onset of the pandemic. For example, drug markets were altered, people experienced increased isolation, and community services were disrupted [17–20]. Further, the pandemic also disrupted drug markets, removing trusted people who sell drugs, and increasing risks associated with buying drugs from unknown sources [115]. There may also have been changes in harm reduction since the interviews were conducted before the COVID-19 pandemic, such as increased access to naloxone and fentanyl test strips. Our interview guide had the following limitations: First, although our guides were harmonized, there were differences at each research site (e.g., recruitment criteria, community drug use trends) that could have influenced variation in responses. Second, the guide did not ask specific behavioral harm reduction questions. Third, interview probes to explore harm reduction as it pertained to fentanyl use were included only with the question regarding injection drug use, which could have resulted in missing information about fentanyl use from people who did not inject drugs and having less nuance regarding other harm reduction details. Fourth, although we asked about local conditions in the interviews, we did not have probes specific to geography and harm reduction. Thus, few geographic differences emerged in participants’ discussion of harm reduction strategies. Finally, given the predominance of community engaged research and sampling methods and the partnership with harm reduction organizations, study participants who were engaged in harm reduction services and practices may have been overrepresented.

## Conclusion

This study provides nuanced insight into harm reduction strategies adopted by people who use drugs in a large, regionally diverse United States rural sample. The rural landscape in the United States is incredibly diverse, and variations in geographical, economic, and social factors can lead to significant differences between different rural regions. In the context of our paper, disparities in infrastructure, resources, and access to essential services are notable. However, few regional differences emerged in participants’ discussion of harm reduction strategies. This paper finds that rural people who use drugs experience different risk environments [116] compared to their urban peers and points to the importance of understanding how local contexts shape harm reduction approaches. Many harm reduction strategies have emerged as community responses to protect oneself and others from a fatal overdose, particularly in locations where the absence of formal prevention efforts led by local, state, or federal agencies are limited or missing altogether. Multiple harm reduction strategies were often used in tandem and influenced drug use behaviors.

### Supplementary Information

Supplementary file 1

## Data Availability

The data from this study are available from the corresponding author upon reasonable request.
