# Supplementary material for: Strategies used to reduce harms associated with fentanyl exposure among rural people who use drugs: multi-site qualitative findings from the rural opioid initiative
Source: Harm Reduct J. 2024 Aug 24;21:154. doi: 10.1186/s12954-024-01062-2 (PMC11344336; doi:10.1186/s12954-024-01062-2)
Supplement: Supplementary file 1 [file 12954_2024_1062_MOESM1_ESM.docx]

***Interview guide objectives: This interview guide is for PWID. The goal of the interview guide is to gain a better understanding of the context of opioid and other drug use, injection drug use, service utilization, and barriers to services in drug-using or PWID populations.***

***Note for interviewers: Probes for each question are flexible. We would like to use similar probes across all sites, but you can adjust the wording, order, etc. as you see fit – they are a guideline for topics to explore.***

**Intro: Thank you so much for talking with me today. As you know, we’re interested in learning more about drug use in [County name], so I have some questions for you. Everything you tell me will be kept confidential and we will not share your name with anyone besides study staff. Stop me at any time if you have any questions for me as we go through, if anything is unclear, or if you would prefer to skip a question.**

**We would like to hear about your experience with opioids and other drugs, so that we can help develop programs and policies that may make services that you actually want to use more available to you. Your participation may help to make things better for people in your situation, and those who come along after you – so we appreciate the time that you are taking to talk to us.**

**Any questions before we begin?**

**Background/Intro**

1. I would like to start by getting to know you a little better.

Probes:

1. *Where did you grow up?* **[If not from the area]**: *How long have you been in this area?*
2. *Tell me about your family and friends.*
   1. *Who do you get help or advice from, when you need it?*
3. *How do you support yourself, financially?*
   1. **[If they work]**: *What do you do for work?*
4. *Tell me about where you live right now? How happy are you with your living situation?*

2. How has this area changed while you have lived here?

3. How has drug use in this area changed while you have lived here?

Probes:

1. *Tell me about any changes in the number of people using drugs to get high?*
2. *Tell me about any changes in what people are using?*
3. *Tell me about any changes in the kinds of people who are using?*
4. *Tell me about reasons you think these changes have happened?*

**Drug Use – History and Current Use**

**Now I’d like to ask you some questions about drug use.**

4. To begin with, tell me about the first time you used a drug aside from alcohol to get high – it could be pain pills, marijuana, or something else.

Probes:

1. *How old were you?*
2. *Who were you with? Where were you? Where did you get the drugs from?*
3. *How was the drug used? (e.g., Swallowed? Snorted? Smoked? Injected?)*

**[For opioid groups]: If the participant has not yet discussed opioid use:**

5. Tell me about any experiences you have had with using an opioid – like a pain pill or heroin – to get high? If yes:

Probes:

1. *How old were you, the first time?*
2. *Who were you with? Where were you? Where did you get the drugs?*
3. *How has your opioid use changed since you first started?*

6. Tell me about the first time you injected a drug.

Probes:

1. *How old were you?*
2. *Where were you? What drug did you use? Who, if anyone were you with? Who prepared it? Who injected you?*
3. *Why did you do it? What was going on in your life at the time?*

7. What drugs are you injecting currently?

**[Note: probes a-e below are for each substance mentioned]:**

Probes:

1. *Tell me about the most recent time that you used it. Where did you get it from? How much did you use?*
2. *Who else was there? Where were you? Is this your ideal place? Why did you use drugs there?*
3. *Whose syringe did you use?* **[if not their own]:** *Who used it before you did? Who used it after you did?*
4. *What did you do, if anything, to protect yourself from harms? (e.g., things like HIV, HCV, overdose, or abscesses?)*
5. *How has your use of this drug changed over time?*

8. You mentioned that you inject **[list all substances mentioned from question 7].**

Probes:

1. **[If haven’t mentioned fentanyl]:** *Have you ever used any drugs containing Fentanyl? Did you realize before or after you took the drug that it contained Fentanyl? Were you seeking a drug that contained Fentanyl, or were you unaware?*
2. *How are the drug preparations different across the drugs (e.g., pills, heroin, fentanyl, methamphetamine, cocaine, etc.)?*
3. *What type of equipment do you use for different drugs? Is the amount of water you add different?*
4. *Do you need to inject more or less frequently depending on the drug? Can you explain?*
5. *Can you tell me any instances when you have injected pills? Can you describe the type of pill? How does the type of processes you use differ from powder?*

**Risk Behaviors:**

9. Now I’ll ask about your experience with overdosing, which includes if you passed out, turned blue, or stopped breathing from using drugs. Have you ever overdosed? **[If yes]:** Tell me about the most recent time that you overdosed.

Probes:

1. *What happened?*
2. *Where were you?*
3. *Were you alone or with others? Who?*
4. *What did people do? Was EMS or 911 called?*
5. *Were you taken to a hospital? Are people concerned about being arrested if 911 is called for an ambulance?*
6. *Was Narcan/naloxone used? If yes, who administered the Narcan/naloxone first?*
7. *What drug(s) were you using?*

10. Tell me about your most significant experience with someone else overdosing? **[If unclear:** In other words, the experience that affected you the most?]

Probes:

1. *Where were you?*
2. *What did people do? What did you do? Was EMS or 911 called?*
3. *Was Narcan/naloxone used?* **[If yes]:** *Who administered the Narcan/naloxone first? Do you currently have Narcan/naloxone with you or at home? If you wanted to get Narcan/naloxone, do you know how to get it?*
4. *What drug(s) were involved?*

**Sexual Partners/Behaviors**

**Next, I’d like to ask you some questions about sex, your sexual partners, and things like condom use. Again, you can choose not to answer questions if you don’t want to.**

11. Tell me about any sexual relationships you currently have*.*

Probes:

*a. How many partners do you have? How long have you been involved with these partners?*

*b. What do you do, if anything, to protect yourself from STDs?*

*c. How often do you use condoms in these relationships?*

1. **[If condoms always used]**: *Tell me the reasons you (your partner) use a condom.*
2. **[If condoms inconsistently used]**: *Tell me about the reasons when a condom is used versus when one is not used.*
3. **[If condoms not used]:** *Tell me the reasons that you (your partner) do not use a condom.*

12. Men and women often exchange sex for things like drugs, housing, food, and other things. Tell me about any experiences you have had with exchanging sex (either yourself or a partner)?

Probes:

1. **[If yes]:** *Tell me about the people that you do this with? Are they men, women, or both?*
2. *What do you do, if anything, to protect against STDs? Against pregnancy?*
3. *Is this type of exchange what you would like to do? If not, what makes it difficult to do what you’d like to do?*

**Interaction with Law Enforcement/Laws and Policies**

**Now I’d like to ask you some questions about your interactions with police (local police, sheriff deputies, state police, DEA).**

13. Tell me about the last time that the police stopped you.

Probes:

1. *What were the reasons that they stopped you? Where were you? What were you doing?*
2. *How did they treat you? What happened in the end?*
3. *Tell me about any experiences you have had with being beaten by the police?*
   1. *What happened?*
4. *Tell me about any times you called the police for help? If so: Tell me about the last time you called the police for help. How did they respond? What were the reasons that you called them?*
   1. *How did they treat you? What happened in the end?*
5. *What do you think about the police, generally?*
6. *Can you tell me about any experiences in jail?*
   1. *What was/were the charge[s]? How were you treated for withdrawal? Did you get any substance use disorder treatment in jail?*
   2. *How was the transition after being released, in terms of your drug use? What happened?*

**Sometimes, state laws and policies just aren’t communicated well to people. I’d like to ask you a few questions about state laws and policies related to drug use.**

14. Tell me what you know about the state’s laws related to possession of drug paraphernalia?

15. Tell me what you know about the state’s laws related to getting or using naloxone (Narcan)? About calling 911 if someone overdoses?

**Services/Healthcare**

**Now I’d like to talk to you about your experiences with health providers, and other community services. To start, I’d like to ask about how you get healthcare and what your experience has been.**

16. Do you have health insurance?

Probes:

1. **[If yes]:** *What kind?*
2. **[If no]:** *What do you do if you’re sick or injured?*

17. How do you decide when it’s time to go to a health care provider?

18. Tell me about your most recent interaction with any doctor or other health care provider.

Probes:

1. *How did you get there?*
2. *How did you feel about the provider? (hint: comfort level, communication style)*
3. *How, if at all, did the topic of drug use come up?*
   1. **[If drug use was discussed]:** *How did the conversation go? What topics did you discuss? Did they discuss the possibility of substance use treatment? What did you like about the conversation? What didn’t you like about it?*
   2. **[If drug use was not discussed]**: *Would you have wanted to talk with your provider about drugs? What kept you from discussing it? What would you have wanted to say or ask?*
4. *Is this typically where you go to seek care?* **[If yes, move on. If no]:** *What are the reasons you chose to go this place versus your normal place? How does this differ from your normal place?*

19. Have you ever decided that you needed care, but didn’t go? Tell me about the reasons you didn’t go.

Probes:

1. *Insurance?*
2. *Transportation?*
3. *Could not make an appointment?*
4. *Afraid/concerned about how the doctor would treat you?*

20. Tell me about any experiences you have had being tested for HIV, hepatitis, or sexually transmitted diseases (such as syphilis, gonorrhea, chlamydia, herpes).

**[Note: probes a-f should be asked for each separately: Hepatitis, HIV, and other STDs]**

Probes:

*a.* **[If tested]:** *What are the reasons that you got tested? Did you ask for the testing, or was it automatically offered to you?*

*b. Where did you get tested? What are the reasons that you went to that specific place?*

*c. What type of test did they give you? How long did it take? How were you treated by health care providers?*

*d. Who told you your results? Tell me about any resources or treatment that they connected you to?*

*e. If you tested positive, have you ever sought treatment from a provider? Tell me about that experience?*

*f.* [**If never tested]:** *Tell me about any thoughts you have had of getting tested. What, if anything, is keeping you from getting tested? What might make it easier for you to seek testing?*

*g. Are you aware of HIV PrEP?*

*i.* **[If yes]:** *Would you be interested in using it? Why or why not?*

*ii.* **[If no]:** *If a daily pill or monthly injection were available that could protect against HIV infection, would you be interested in using it? Why or why not? Would your friends who inject with you be interested?*

21. Tell me about any times you went to a clinic or got treatment that could help you with drug use? (e.g. detox center? recovery center? Primary care office?)

Probes:

1. **[If Yes]:** *Tell me about your experience.*
   1. *What type of clinic was this? What did they offer—talk therapy, faith-based services, Medication assisted treatment—methadone, etc.? Did options include inpatient or outpatient services? Short-term or long-term services?*
   2. *Where was this? How far of a drive?*
   3. *How long ago was this?*
   4. *How well were you able to get the services you needed?*

*b. Tell me about any times you* ***wanted*** *to access a detox center or drug treatment program, but weren’t able to. What got in the way?*

1. *Insurance?*
2. *Transportation?*
3. *Hours the center or program was open?*
4. *Waitlist/availability?*
5. *Affordability?*
6. *Afraid/concerned about how staff would treat you?*

c*. Tell me about any other experiences you have had receiving drug-related services.* *What kind of clinics or providers did you go to?*

*d. Tell me about your interest in accessing methadone or buprenorphine treatment in the future.*

- 1. **[If interested]:** *Why are you interested? Is there anything that might make it easier for you?*
  2. **[If not interested]:** *What makes you feel that way?*

22. Tell me about any times you got a needle or syringe from a syringe exchange program?

Probes:

1. *Tell me about your experiences getting needles or syringes from a syringe exchange program? This could include you going yourself, or getting them from someone who went to a syringe exchange program.*
   1. **[If getting from someone else]:** *Why didn’t you go yourself? Awareness/hours/access/concern about how program staff would treat you?*
2. *How did you first hear about it?*
3. *How often do you use it? How many syringes do you typically turn in and how many can you get?*
4. *What do you like about it? Tell me about any challenges to using it? What other services do you think it should offer? How could it be made better?*

23. What other services would you be interested in receiving?

Probes:

*a. What locations would you prefer to go to?*

24. We have talked about many things today. I really appreciate your willingness to share your thoughts. Is there anything else that you feel that I should know or that we haven’t covered but you feel is important for us to know?

**Conclusion: Thank you so much for talking with me today – we really appreciate it. If you have any concerns, please don’t hesitate to reach out at the number provided on the consent form!**
